# Supplementary material for: Characterization of Palladium Nanoparticles Produced by Healthy and Microwave-Injured Cells of Desulfovibrio desulfuricans and Escherichia coli
Source: Nanomaterials (Basel). 2019 Jun 5;9(6):857. doi: 10.3390/nano9060857 (PMC6630224; doi:10.3390/nano9060857)
Supplement: Supplementary file 1 [file nanomaterials-09-00857-s001.pdf]

Supplementary Materials

Characterization of Palladium Nanoparticles Produced by Healthy and Microwave-Injured Cells of *Desulfovibrio desulfuricans* and *Escherichia coli*

Jaime Gomez-Bolivar [1,*], Iryna P. Mikheenko [2], Lynne E. Macaskie [2] and Mohamed L. Merroun [1]

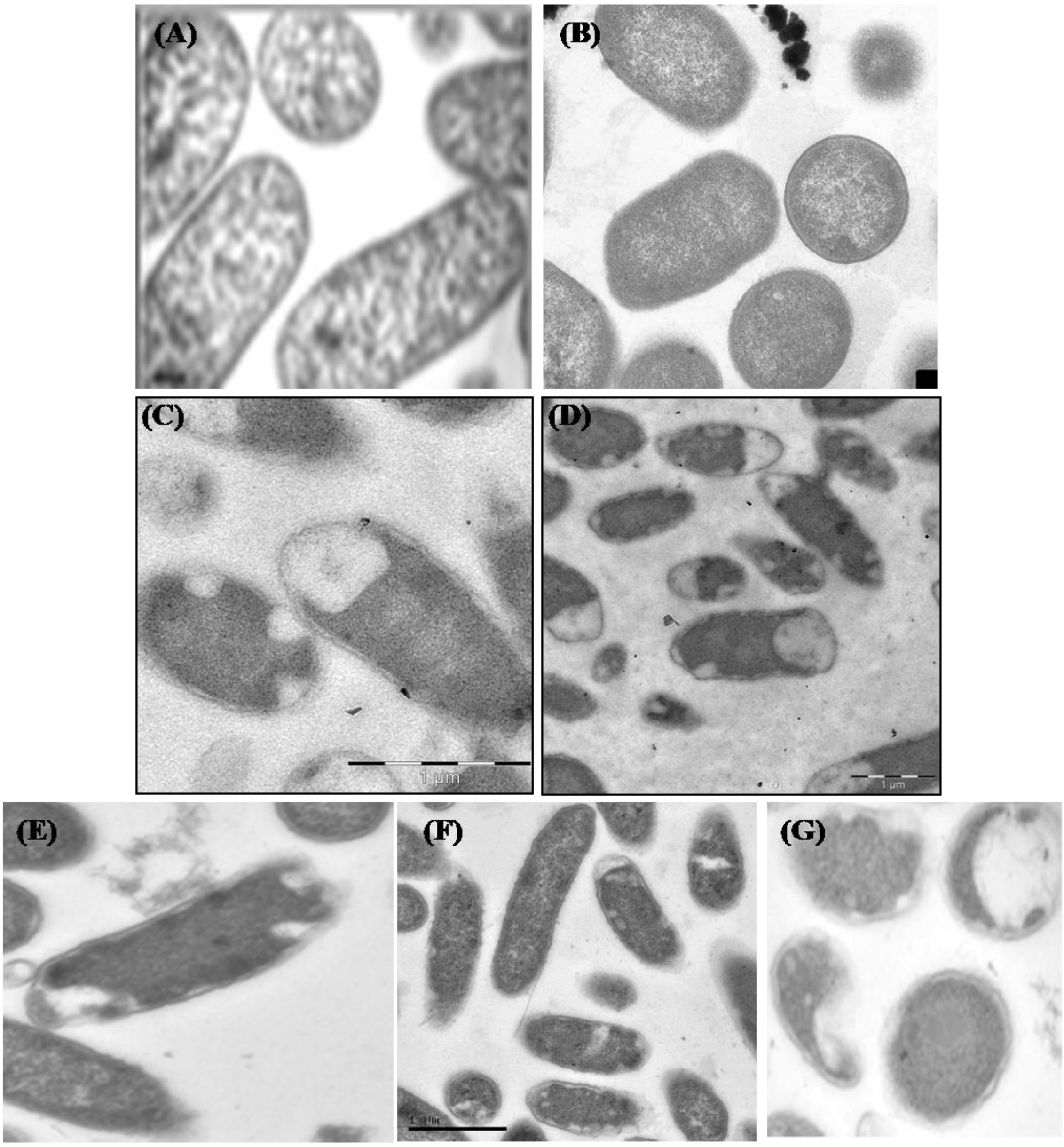

Figure S1. Effect of RF irradiation (microwaves) on cells of *E. coli* and *D. desulfuricans* in the absence of microwave treatment (A,B, respectively) and following exposure to MW in short bursts with cooling (C, D, respectively) (this study: see text). A companion study was done (E,F,G) which applied RF radiation at 2W

(E),4W (F) or 8W (G) (20 min) using purpose-built equipment to decouple the thermal and electromagnetic components. The equipment was developed in house at C-Tech Innovation Ltd. via adaptation of commercial equipment. The *E* (electric) component of electromagnetic radiation produces heating in dielectric (non-conducting) materials so it is important to exclude heating from the sample by isolating the *H* (magnetic) component. This was achieved using a series LC circuit tuned with an external matching box. This generated a magnetic field inside the induction coil and an electric field in the air gap between the two conducting plates. The sample vial, placed in the centre of the induction coil, was exposed to a magnetic field for the desired time. After exposure, the final temperature of the bottle was checked and absence of heating confirmed. The samples were treated by exposure inside a solenoid coil as the electromagnetic field within the coil will be almost entirely magnetic; consequently, as the magnetic susceptibilities of the components of the sample being treated (glass, water, bacteria) are very low at this frequency there would be minimal heating of the sample, allowing long treatment times. The total dose in the two studies was similar: The commercial equipment (this study) delivered 300W which was applied for 30 sec (9,000 'units of stress''). In the purpose-built equipment the cells were treated with 2-8 W over 20 min (up to 9,600 'units of stress'); the power level can be considered as a rate (power = energy per second; 8W; 20 x 60 sec) and a dose as rate x time of the integral of rate over time.

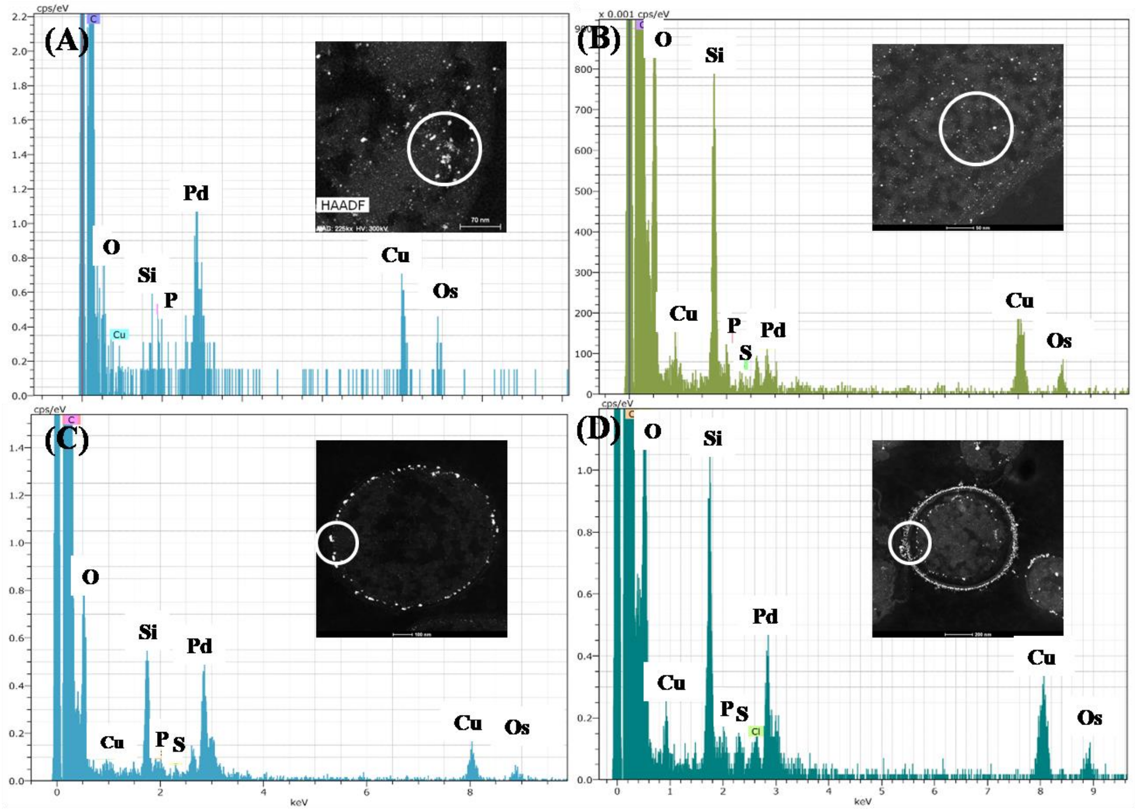

Figure S2. HAADF–STEM–EDX analysis of Pd nanoparticles (circled areas shown) with phosphorus (P) and sulfur (S) of untreated cells of *E. coli* MC4100 (A) and *D. desulfuricans* (C), 30 second MW treated cells of *E. coli* MC4100 (B) and *D. desulfuricans* (D). Silicon is from the oil diffusion pump of the column of the TEM system, copper is from the TEM grid and osmium from the staining
